# Unlocking the Potential of Type 2 Diabetes Mellitus Remission

**DOI:** 10.7759/cureus.48704

**Published:** 2023-11-12

**Authors:** Prakriti Sharma, Swarupa Chakole

**Affiliations:** 1 Community Medicine, Jawaharlal Nehru Medical College, Datta Meghe Institute of Higher Education and Research, Wardha, IND

**Keywords:** tirzepatide, very low-calorie diet, bariatric surgery, twin cycle hypothesis, diabetes remission

## Abstract

Diabetes was considered manageable but not curable, but now there are methods by which diabetes can be reversed. The twin cycle hypothesis provides a bird's eye view on the pathogenesis of the onset of diabetes, which is necessary to understand for reversing the disease: it states that diabetes occurs due to a substantial accumulation of fat in the pancreas and liver, impairing the β-cell function. Thus, we can infer that diabetes and obesity are two aspects of the same problem. Thus, the key to diabetes reversal is to reverse obesity. Bariatric surgery has shown promising results. Diabetes remission can also be achieved by reducing calories. A very low-calorie diet (VLCD) results in rapid weight loss. β-cell recovery is possible if early-diagnosed individuals are treated with intensive insulin therapy. New drugs like liraglutide and tirzepatide also have the potential for diabetes reversal. Thus the age-old myth of diabetes being incurable is proven wrong.

## Introduction and background

Type 2 diabetes mellitus is a metabolic disorder of abnormal glucose homeostasis. The fundamental problems of diabetes are insulin resistance and impaired insulin secretion. Environmental factors that affect diabetes include being overweight and being sedentary. Genetic predisposition may also play a pivotal role. Type 2 diabetes is a gradual illness that emerges in the elderly due to impairment in the working of β-cells and the development of resistance resulting from overindulgence in calories for years [1]. This results in unconventional fat storage and dysfunction in the lipid cycle. Adult diabetes mellitus develops when 40-60% of the working β-cell mass vanishes [2]. Prolonged hyperglycemic conditions can lead to severe complications. This article discusses achieving a normoglycemic state in people with type 2 diabetes mellitus via metabolic surgery, significant lifestyle modification, and therapeutic interventions. Some people who had bariatric surgery reached diabetes remission due to decreased calorie intake along with the role of gut hormones [3]. Obesity is a recognized risk factor for the development of comorbid conditions such as cardiovascular disease, and type 2 diabetes mellitus. Today, Roux-en-Y gastric bypass (RYGB), sleeve gastrectomy (SG), and adjustable gastric banding are the most popular and commonly performed bariatric surgeries (BS) [4,5]. It is also found that reduced weight via a reduced-calorie diet reduces accumulated adiposity in the pancreas and liver and reverses insulin insensitivity. Recently discovered drugs like liraglutide and tirzepatide are leading to markable changes in the management of diabetes.

## Review

Methodology

**Figure 1:**
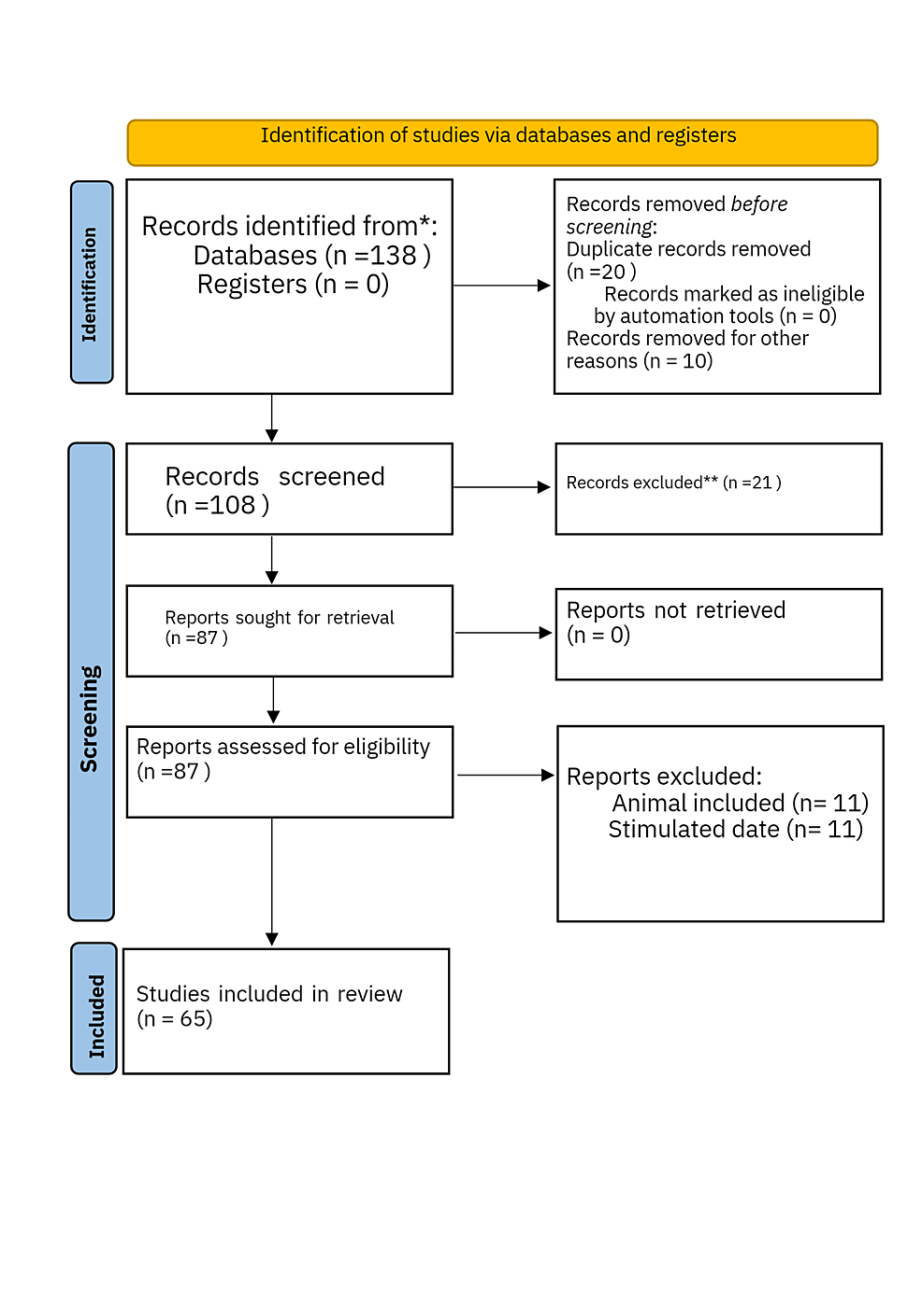
PRISMA flow diagram *20 records removed due to duplication **21 records were excluded due to lack of correlation

We conducted a methodical search across PubMed, Google Scholar, and Science Direct in May 2023 using main words such as “diabetes type 2” and “diabetes remission” (((diabetes type 2* [Title/ Abstract]) OR (adult onset diabetes [Title/Abstract])) OR (diabetes mellitus [Title/Abstract])) OR (“diabetes” [MeSH Terms]) AND ((“remission”* [Title/Abstract] OR (“reversal” [Title/Abstract])) OR (“remission” [MeSH Terms]). Additionally, we researched for key references from bibliographies of the appropriate studies. All relevant studies, including review articles, meta-analyses, and original research, were carefully arranged, and preference for the most recent study was given. The flow diagram for PRISMA is shown in Figure 1 below.

Pathogenesis of type 2 diabetes mellitus

To see the big picture, we have to put together the puzzle pieces in the proper order. Similarly, to understand the reversal of diabetes, all the elements of pathogenesis must be understood. Type 2 diabetes is a long-standing disease that has multifactorial causation. Despite the influence of genes and habitat variables, metabolic processes are crucial in the development of the illness. In people enduring the agony of type 2 diabetes, there is compromised glucose homeostasis, which is caused by reduced insulin secretion or insulin resistance at different levels such as in the muscles, liver, adipocytes, or by anomalies in glucose uptake. The pathogenesis of type 2 diabetes mellitus is explained through "the twin cycle hypothesis", first introduced in 2008 [6].

Twin Cycle Hypothesis

This hypothesis indicates that type 2 diabetes mellitus is a consequence of a surplus supply of fat to the liver and pancreas, which results in functional impairment of both organs. The excess liver triglycerides will cause a plunge in the hepatocytes' insulin reciprocation, which leads to insulin resistance. Due to insulin resistance, gluconeogenesis is brought to a halt, causing high plasma glucose, and due to the physiological feedback system of the human body, insulin level in the blood also increases. As a result, the excess glucose is converted to fat in the liver, causing hepatic steatosis, which insulin encourages [7]. The raised plasma triglycerol level also influences the amount of liver fat [8]. This triggers the ruthless hyperlipidemia and hyperglycemia cycle. Due to repeated activation of the liver cycle, the liver starts to export very low-density lipoprotein triglycerides, increasing its level in the blood. Soon the threshold of fat storage in the adipocytes will be reached, and abnormal fat storage in the pancreas. The threshold depends on the individual [9]. This will push the pancreas cycle. Excess exposure to triglycerides, fatty acids, and toxic metabolites will reduce insulin secretion. When enough β-cell are damaged, type 2 diabetes is set. The fat storage is redirected to the liver from adipose tissues through de-novo lipogenesis. The triglycerides and fatty acid get metabolized, which leads to the formation of toxic by-products that, in turn, causes insulin resistance. To counterbalance the opposition, the body produces more insulin, promoting lipogenesis. The pancreatic cycle is activated due to the increased export of VLDL. The pancreas takes up a high number of fatty acids and stores them. This causes the diminution of β-cells. This ruthless cycle causes hyperglycemia and high insulinemia [10,11]. Figure 1 below describes the concept of the twin cycle hypothesis.

**Figure 2:**
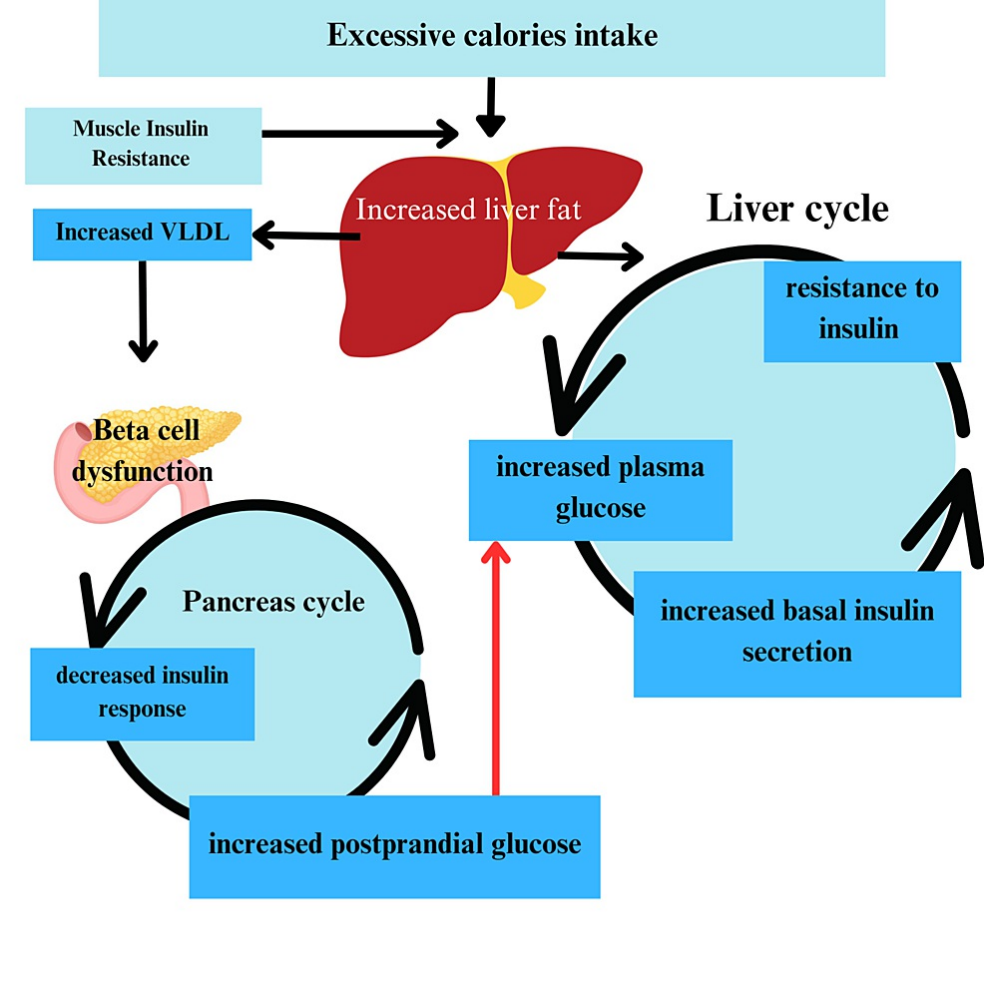
Twin Cycle Hypothesis VLDL: Very-low-density lipoprotein The figure is self-created by the author.

New Aspects of Insulin Non-Compliance

In muscles, the quickest sign of type 2 diabetes is the onset of skeletal muscle insulin insensitivity [12]. One astonishing finding is that mice lacking skeletal muscle insulin receptors do not develop diabetes [13]. Because of the muscle glycogen synthase PPP1R3A variant in some humans, they could not store glycogen [14]. In the long run, this causes increases in blood glucose levels. It's an old concept that the risk of diabetes is preceded by insulin resistance. Recent research proves that the possibility of diabetes is decided first by extrahepatic hostility towards insulin [15]. In the liver, before diabetes develops, it has been seen that the liver has uniformly deposited unhealthy fat [16]. In patients receiving insulin therapy, the dose of insulin depends upon the amount of unwanted fat in the liver [17]. Excess consumption of calories causes hepatic fat storage. It has been shown that excess sucrose for a period of three weeks causes a 30% hike in the fat content of the liver [18]. The extra sugar is converted to fatty acids. Malonyl-CoA generated during de novo lipogenesis prevents fatty acid transport into mitochondria [19]. Steatosis of the liver is also due to the reduced ability of hepatocytes to oxidize fat [20]. Since the fatty acids are not oxidized to energy, as in normal circumstances, they combine with glycerol to form diacylglycerols. A high level of diacylglycerol activates the protein kinase C epsilon type, blocks the signalling route between the insulin receptor and substrate, and impairs the activity of the hormone insulin. This heightens the insensitivity to insulin [21].

New Aspects of β-cell Defect

β-cell defects in type 2 diabetes have two components: reduction in the count of β-cells and gradual impairment in its function to boost insulin production in response to blood sugar [22]. Recently, it was discovered that when β-cells show a change in gene expression, they retrogress to an immature form, and in some cases, they transdifferentiate to other islet cell types [23]. Due to excess fatty acid in the pancreas, pyruvate cycling and pyruvate dehydrogenase are inhibited. Since glucose oxidation is prevented, there is a decrease in adenosine triphosphate production, which is necessary for insulin secretion [24]. β-cell loss increases proportionately to the duration of diabetes [25]. The loss of β-cells occurs due to apoptosis. This apoptosis is influenced by prolonged exposure to fatty acids [26]. In addition to apoptosis, β-cell mass is reduced due to loss of differentiation. The term "loss of differentiation" first appeared in the late 1990s [27].

The potential of diabetes reversal

Currently, type 2 diabetes mellitus is a universal issue. It is anticipated that 700 million people worldwide will have diabetes by 2045. Society now regards diabetes as treatable but not curable, but these concepts are now changing due to recent discoveries in the field of diabetes reversal. Now there is a cure for diabetic patients too through weight loss and decreasing calorie intake. For the interest of humanity, we, therefore, need to comprehend this transformation in type 2 diabetes mellitus [28].

Diabetes reversal is also recognized as diabetes remission. The criteria to pinpoint the reversal is still debated. A systemic review on remission has identified more than 100 definitions of diabetes remission. Still, the most proper way is through haemoglobin A1c, which should be below 6.5% measured after three months of cessation of anti-diabetic drugs and at least six months after pursuing a healthy lifestyle [29,30]. This definition, however, has certain limitations as it restricts our viewpoint around glucose control and ignores the value of weight loss, changes in vessels, and the heart. Glycosylated haemoglobin below 6.5% does not imply that there will be no complications, as cardiovascular risk is rooted even before the onset of diabetes [31,32]. Still, this criterion is widely used because it has high reproducibility by international standards [33]. According to recent studies, continuous glucose monitoring systems like time in the range are also used as strategies for diabetes remission [34].

Metabolic surgery for diabetes reversal

Obesity and diabetes go hand in hand. Present-day studies show that around 1.9 billion adults are overweight, and nearly 2.8 million deaths occur due to obesity-related problems like diabetes and cardiovascular problems. In India, more than 135 million people were categorized as obese. According to ICMR-INDIAB 2015, the reported prevalence rate of obesity alters from 11.8% to 31.8%, and abdominal obesity varies from 16.9% to 36.3% [35]. A study shows that over 462 million individuals were affected by type 2 diabetes in 2017, corresponding to 6.28% of the world’s population, thus making the prevalence rate of type 2 diabetes around 6059 cases per 100,000 people. Over 1 million deaths per year are due to diabetes alone, making it the ninth leading cause of mortality [36]. Diabetes mellitus is now the ninth leading cause of death worldwide, according to WHO. It is anticipated that by 2045, 700 million people will be having diabetes [37]. Even after significant advancements in medical science and pharmacotherapy, the incidence of diabetes is not decreasing, and the interventions are failing. The cause of the failure of this intervention may be rapid urbanization. So these prevention measures are overshadowed by the rising incidence of diabetes.

The global burden of type 2 diabetes mellitus and adiposity is a two-sided coin. Because of the hike in the prevalence of this burden, surgical intervention was introduced around 1990, and it was observed that diabetes reversal is possible after weight reduction surgery. Since its introduction, diversity in weight reduction procedures increased. Throughout history, approximately 50 bariatric procedures have been described [38]. It includes gastric banding, banded gastroplasty, sleeve gastrectomy, Roux-en-Y gastric bypass, and biliopancreatic diversion. The most common weight loss surgery is gastric bypass surgery following sleeve gastrectomy and adjustable gastric band. The Federation of the Surgery of Obesity and Metabolic Disorders (IFSO) published a report in 2019 claiming that 833,687 bariatric surgeries were performed globally. The surgical procedure also reduces the requirements for diabetic medications and insulin administration. Up to 60-80% of cases showed remission of diabetes [39]. The probability of diabetes reversal is twice as high with Roux-en-Y gastric bypass in contrast to gastric banding [40]. 

The Mechanism Behind Weight Reduction After Surgery

Notably, remission of diabetes usually occurs after a short duration of the surgery (a couple of days to a couple of weeks); within this duration, weight loss has not even started. The mechanism behind the astonishing results of bariatric surgery includes three methods that result in decreased weight: gastric restriction, malabsorption, or both. A small gastric pouch and an outlet obstruction are created in gastric restriction. The small pouch is designed for replication of satiety and outlet obstruction, delaying emptying time. Reduced absorption occurs as a result of the reduced gut or remote blending of bile and pancreatic contents with food, leading to malabsorption. The gastric bypass procedure includes both of these mechanisms, as diabetes and being obese are strongly correlated with one another, so weight reduction is a prime concern for patients. The non-surgical intervention for weight reduction is not that effective as it has a short-term effect of nearly 5-10% of body weight reduction at best up to 18.8% [41]. Pharmacotherapy and lifestyle changes guarantee only 2.6% to 8.8% weight loss [42]. Contrary to this, bariatric surgery leads to 50% to 75% loss of excess fat [43]. Weight reduction is maintained longer; studies show it can be secured for 16 years [44,45]. 

Aftermath of Bariatric Surgery: The Action of Gut Hormones

There will be a reduction in the patient's calorie intake postoperatively because of decreased hunger and increased satiety [46]. Whenever there is diet-induced weight loss, there are increases in the amount of ghrelin, better known as the "hunger hormone", and "satiety hormones" are decreased [47]. In spite of this, there is an increase in the amounts of glucagon-like peptide-1 (GLP-1), peptide YY (PYY), and oxyntomodulin (OXM), which are "satiety hormones" after bariatric surgery [48,49]. Immediately after gastric bypass, there is an increased response of β-cells to a meal, along with raised GLP-1 secretion [50].

Criteria for Patient Selection

In a 1991 statement, the National Institutes of Health (NIH) laid out the selection guidelines. They stated that surgery should be considered for an individual whose body mass index (BMI) exceeds 40 and whose lifestyle is severely impaired due to their weight. They also recommended that non-surgical treatment should be preferred. They also gave another criterion that bariatric surgery can be done in an individual with 35 and 40 BMI, provided that they suffer from high-risk conditions like diabetes mellitus, cardiovascular disorders, hyperlipidemia, and obstructive sleep apnoea [51].

Complications After Bariatric Surgery

Many complications may arise after any bariatric procedure, and the most notable ones are nutrient deficiencies and gastrointestinal pathologies. As gastric restriction and malabsorption are the mechanisms behind the remission of diabetes by reversing obesity, there are chances of deficiency of nutrients. The stomach and parts of the small intestine have a pivotal role in absorbing vitamin b12, iron, and calcium. Iron deficiency is the most common micronutrient deficiency after gastric bypass surgery. Thus proper supplementations should be provided to avoid the development of anaemia. Protein deficiency in the initial duration may be tougher to manage as it needs to be taken through meals, and the quantity of food intake is reduced after surgery. This protein deficiency may present as alopecia. Therefore we must have an eye on protein levels during the initial months. The pathological complication may include vomiting and gastroesophageal regurgitation due to the obstruction. Some patients who undergo gastric restriction may not limit their food consumption, which does not fit the gastric pouch and, consequently, expands. This also leads to the failure of weight reduction. Even after this, when surplus food intake continues, the oesophagus will act as a reservoir and give an achalasia-like appearance and may be associated with regurgitation and nocturnal aspiration [52].

Diabetes reversal through calorie restriction

In today's era of urbanization and development, the need for an increasing number of people to walk and shed sweat for work is next to negligible, and the sedentary lifestyle has made it easier for diabetes to set in. Thus, behaviour change, exercise, and calorie intake restrictions are needed to reduce excess weight. One can reduce weight by following a low-calorie diet (LCD) that permits the intake of 1200-1500 kcal/day [53]. Another option is a very low-calorie diet that allows 400-800 kcal/day [54].

Very-Low Calorie Diet

The popularity of a very low-calorie diet (VLCD) has been rising since its introduction in 1970. It is very effective because it produces rapid weight loss of approximately 20-30% in 12-16 weeks. The VLCD regimen usually involves two phases, and the initial phase is in which all food is replaced by a liquid diet formula that will provide 400-800 kcal/day for 12-16 weeks. Later, solid food is introduced again in a very structured manner. The liquid constitutes 50-60% kcal from maltodextrins and sucrose to prevent ketosis, the correct number of fatty acids, and protein to avoid lean muscle mass loss. VLCD led to significant weight reduction and maintained remission in 45-60% of patients within 12 months. Most of them achieve it before 24 months at most. Throughout the diet course, vitamin and mineral supplements are advised to be consumed to avoid deficiencies. Due to the consumption of oral hypoglycemic drugs during the diet, there is a greater risk of hypoglycemia. Thus, close supervision is required, and dose modification or discontinuation is done. It has been recently demonstrated that sustained diabetes reversal can occur with VLDL approaches [55].

Diabetes remission through drugs

Through the development of science, we have discovered many hypoglycemic drugs that offer many beneficial effects other than maintaining glucose levels. Intensive insulin treatment in newly diagnosed diabetes patients has proven its worth in terms of remission. According to this study, newly diagnosed individuals with diabetes who underwent regular insulin through subcutaneous infusions for two to three weeks experienced glycemic control and β-cell recovery [56,57]. Their remission after intensive insulin treatment lasted more than two years. The odds of reversal are significantly increased with a shorter period between diagnosis and rigorous insulin therapy [58].

Recently drugs working with unique mechanisms that lead to weight loss have been discovered. The sodium-glucose cotransporter-2 (SGLT2) inhibitor is one of these pharmacological classes. It lowers glucose absorption in a manner that causes glycosuria in the proximal convoluted tubule of the nephron and has a reducing impact independent of insulin secretion and sensitivity [59]. Because of the excretion of glucose through urine, this drug class reduces fat from the visceral organs [60]. We know that a hormone called glucagon-like peptide-1 (GLP1) inhibits glucagon secretion; thus, if GLP1 receptor agonists (GLP1-RAs) are used, it will help in reducing fat storage in the liver [61]. A type of GLP1-RA is liraglutide, which is known to sustain β-cells provided it is used immediately after diagnosis of diabetes [62]. A recently licensed medication with an additive effect on glucose-dependent insulinotropic polypeptide (GIP) and GLP1 is tirzepatide [63]. Tirzepatide has a remission rate of 66%-81% over 52 weeks, depending on the dosage [64]. 

Keeping aside the immense benefits of these drugs, there are also some drawbacks. SGLT2 inhibitors cannot be administered in chronic diabetic patients as there have been cases of ketoacidosis [65]. The GLP1-RAs and tripeptides are administered through injections, which is not a patient-friendly procedure. It causes a psychological burden plus the risk of local reaction. Some patients discontinued these drugs due to gastrointestinal discomfort [66]. These drugs are also expensive, thus limiting the population that could access them.

## Conclusions

The global burden of diabetes keeps on rising. An increase in the number of diabetes patients also means an increased patient count with comorbid conditions like cardiovascular pathologies. Thus interventions on a global scale are needed to combat diabetes. The best course of action for achieving reversal is bariatric surgery, but it is only available to a limited patient population due to its high cost and complications. Diet and lifestyle modifications are a less invasive way of achieving remission status. Intensive insulin therapy in recently diagnosed patients shows recovery in β-cell functioning. New drugs like SGLT2 inhibitors and GLP1- RAs have shown promising results in losing weight, making them relatively safer in achieving remission status.
